# Diversity Profile of Microbes Associated with Anaerobic Sulfur Oxidation in an Upflow Anaerobic Sludge Blanket Reactor Treating Municipal Sewage

**DOI:** 10.1264/jsme2.ME14105

**Published:** 2015-03-28

**Authors:** Azrina A. Aida, Kyohei Kuroda, Masamitsu Yamamoto, Akinobu Nakamura, Masashi Hatamoto, Takashi Yamaguchi

**Affiliations:** 1 Department of Environmental Systems Engineering, Nagaoka University of Technology1603–1 Kamitomioka-machi, Nagaoka, Niigata, 940–2188Japan; 2 Halal Products Research Institute, Universiti Putra Malaysia43400 UPM Serdang, SelangorMalaysia

**Keywords:** anaerobic sulfur oxidation, microbial diversity, next-generation sequencing, UASB reactor, wastewater treatment

## Abstract

We herein analyzed the diversity of microbes involved in anaerobic sulfur oxidation in an upflow anaerobic sludge blanket (UASB) reactor used for treating municipal sewage under low-temperature conditions. Anaerobic sulfur oxidation occurred in the absence of oxygen, with nitrite and nitrate as electron acceptors; however, reactor performance parameters demonstrated that anaerobic conditions were maintained. In order to gain insights into the underlying basis of anaerobic sulfur oxidation, the microbial diversity that exists in the UASB sludge was analyzed comprehensively to determine their identities and contribution to sulfur oxidation. Sludge samples were collected from the UASB reactor over a period of 2 years and used for bacterial 16S rRNA gene-based terminal restriction fragment length polymorphism (T-RFLP) and next-generation sequencing analyses. T-RFLP and sequencing results both showed that microbial community patterns changed markedly from day 537 onwards. Bacteria belonging to the genus *Desulforhabdus* within the phylum *Proteobacteria* and uncultured bacteria within the phylum *Fusobacteria* were the main groups observed during the period of anaerobic sulfur oxidation. Their abundance correlated with temperature, suggesting that these bacterial groups played roles in anaerobic sulfur oxidation in UASB reactors.

Microorganisms are diverse and complex life forms. They play varied roles in the cycles of elements such as sulfur, nitrogen, carbon, and iron, and have an important environmental impact. In the sulfur cycle, sulfur-oxidizing and -reducing bacteria play various crucial roles in different anaerobic environments; they also represent a key element in biological wastewater treatment plants (39). Sulfur-oxidizing bacteria (SOB), in particular, are the main microorganisms contributing to the bioremediation of the sulfide-rich wastewater generated by many industries such as the petroleum, mining, textile dyeing, pulp and paper, food processing and sulfate-containing wastewater treatment industries, as well as by tanneries (19, 39). SOB also play a role in wastewater treatment that involves anaerobic sulfur oxidation in upflow anaerobic sludge blanket (UASB) reactors that are used for the treatment of municipal sewage (29).

SOB generally oxidize hydrogen sulfide, sulfur, sulfite, thiosulfate, and polythionates such as tri-, tetra- and pentathionate to sulfate as the main oxidation product under acidic, neutral, or alkaline environments (32). The oxidation of these reduced sulfur compounds has mainly been attributed to chemolithotrophic and photoautotrophic bacteria (13). Chemolithotrophic bacteria, which are also referred to as colorless sulfur bacteria, use oxygen or oxidized iron under aerobic conditions or nitrate and nitrite under anaerobic conditions as the terminal electron acceptors. As with photoautotrophic bacteria, carbon dioxide is used as the terminal electron acceptor by green and purple sulfur bacteria under anaerobic conditions (12, 39).

Previous studies reported the structures and activities of the microbial communities present in anaerobic bioreactors used to treat various types of wastewater under different parameters (3, 5, 8, 23, 26, 28). However, our knowledge and understanding of the mechanisms underlying anaerobic sulfur oxidation as well as the interactions between microbial communities and environmental components remain incomplete and undefined in some areas. Significant differences exist in the physiology of many bacterial species even though they share certain structural, genetic, and metabolic characteristics.

Therefore, we herein analyzed the abundance, distribution, characteristics, and phylogenetic diversity of the microorganisms existing in a UASB reactor over a period of 2 years in order to gain greater insights into microbial biodiversity and its role in anaerobic sulfur oxidation. This will also eventually contribute novel and additional information to the microbial biodiversity data presently available.

## Materials and Methods

### Reactor operation, sample collection, and water quality analysis

A closed settling compartment UASB reactor with a total volume of 1,178 L and height of 4.7 m was operated at ambient temperature. The hydraulic retention time of the system was set to 8 h. Additional details on the UASB reactor have been described previously (38). The system was fed with raw sewage that had 150 mg-S L^−1^ sodium sulfate initially added and was subsequently maintained at 50 mg-S L^−1^ sodium sulfate throughout the operation. The system was set up at the municipal sewage treatment plant in Nagaoka, Niigata, Japan. Before and after the addition of sodium sulfate, sludge samples were collected from port 5 of the UASB reactor, which is located 1.278 m from the bottom of the reactor, over a period of 2 years of operation and kept in a container containing ice during delivery to the laboratory. The collected samples were then immediately stored at −20°C until required for the microbial analysis. Portable devices were used to measure the temperature and pH by a pH meter (HM-20P; TOA DKK, Tokyo, Japan), oxidation-reduction potential (ORP) by an ORP meter (RM-20P; TOA DKK), and dissolved oxygen (DO) by a DO meter (YSI 58-115V; Xylem, Kanagawa, Japan) on site. A high-performance liquid chromatography (HPLC) system (LC 20-ADsp; Shimadzu, Kyoto, Japan) was used to analyze sulfate, nitrate, and nitrite contents, whereas a HACH water quality analyzer (DR2500; HACH, Loveland, CO, USA) was used for the chemical oxygen demand (COD) analysis. A sulfide analysis was conducted according to the standard methods published by the Japan Sewage Works Association (21), which was briefly described as follows: hydrogen sulfide gas, which was produced after the addition of sulfuric acid into the sample, was absorbed into the zinc acetate solution and produced zinc sulfide. Iodine solution and hydrochloric acid were then added into the solution containing zinc sulfide. Starch was also added into the solution as an indicator, which produced a color change from blue to transparency after titration with sodium thiosulfate to measure the sulfide content.

### DNA extraction, polymerase chain reaction (PCR) and terminal-restriction fragment length polymorphism (T-RFLP) analyses

Genomic DNA was isolated from the sludge samples using a FastDNA SPIN Kit for Soil (MP Biomedicals, Carlsbad, CA, USA) according to the manufacturer’s protocol. The DNA concentration was determined using a NanoDrop Spectrophotometer ND-1000 (Thermo Fisher Scientific, Waltham, MA, USA). PCR was performed using a set of bacteria-specific primers, EUB338f, and fluorescently labeled primers 907r (15) under the following conditions: an initial denaturation step of 94°C for 2 min, followed by 35 cycles of denaturation at 94°C for 30 s, annealing at 50°C for 1 min, and extension at 72°C for 1 min. The final cycle was followed by final extension at 72°C for 4 min. The *Hha*I restriction enzyme was used to digest the PCR products of bacteria 16S rRNA gene fragments that had been purified with a QIAquick PCR purification kit (Qiagen, Hilden, Germany) and were then analyzed through a CEG-2000XL capillary sequencer (Beckman Coulter, Fullerton, CA, USA) as described previously by Hatamoto *et al.* (16). The TRiFLe program was used to perform an *in silico* terminal restriction fragment (T-RF) prediction as described by Junier *et al.* (22), and the predicted T-RFs were then correlated with the sequencing results.

### Next-generation sequencing and data analysis

PCR amplification of the 16S rRNA gene from extracted DNA for sequencing was carried out according to Caporaso *et al.* (7) by using the primers 515F and 806R. Reactions were held at 94°C for 3 min as an initial denaturation step, with amplification proceeding for 35 cycles at 94°C for 45 s, 50°C for 60 s, and 72°C for 90 s. A final extension of 10 min at 72°C was added to ensure complete amplification. The PCR products were purified according to the protocol provided in the QIAquick PCR purification kit (Qiagen) and were then analyzed through a MiSeq sequencer (Illumina, San Diego, CA, USA), which targeted the V4 region of the bacterial 16S rRNA gene (7).

The sequencing data of the 16S rRNA gene were analyzed using Quantitative Insights Into Microbial Ecology (QIIME) software for a microbial community analysis (6). The 100 base reads were cut with quality scores of two or more consecutive base calls below 1e–5. A minimum length of 75 bases was required for inclusion in the analysis and any reads that contained an N character were discarded. Operational taxonomic unit (OTU) picking was performed by clustering the sequence at 97% identity using UCLUST (11) and BLAST against the SILVA database (30). The BLAST matches were chosen based on the E-value (the maximum value was 1e–10), the percentage sequence identity of the alignment between a BLAST match and the read, which had to be greater than or equal to the OTU selection threshold (0.97 here, corresponding to species-like OTUs), and the match that achieved the longest alignment to the read. Chimeric sequences were identified with ChimeraSlayer (14). Alpha diversity was determined using the calculation of observed species, Chao1, Phylogenetic diversity (PD), the Shannon index, the Simpson index, and sampling intensity (coverage) at each sampling depth. Weighted UniFrac, a quantitative measure of beta diversity, was used to perform a principal coordinate analysis (PCoA) in order to determine similarities between samples (27).

### Sequence data accession number

Nucleotide sequence data was deposited to the DDBJ Sequence Read Archive under accession number DRA002293.

## Results and Discussion

### Performance of the UASB reactor

**Fig. 1:**
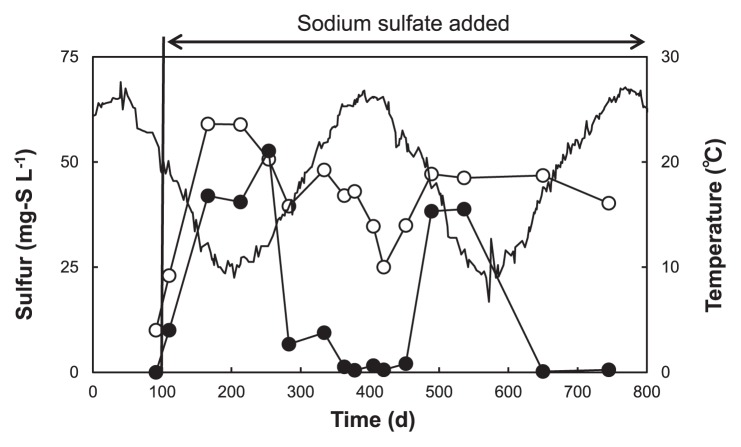
Time course of reduced (○) and oxidized (●) sulfur concentrations and influent sewage temperature (−) of the UASB reactor. Reduced and oxidized sulfur concentrations were calculated from the UASB profile results.

**Fig. 2:**
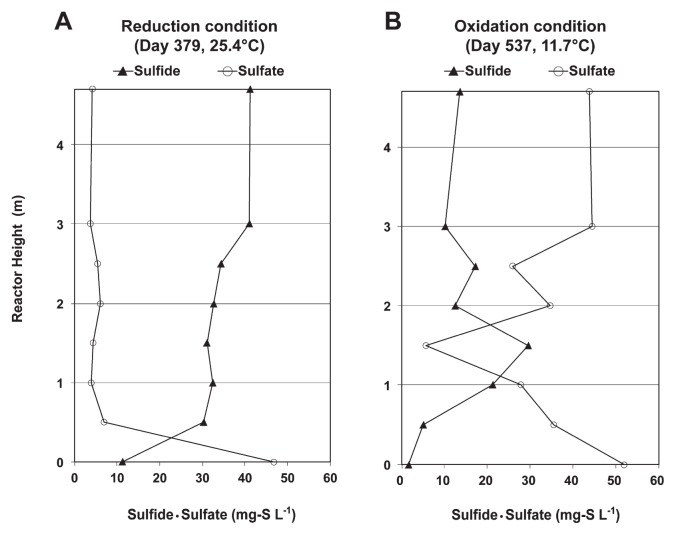
UASB profiles of sulfide and sulfate. A–non-occurrence of anaerobic sulfur oxidation; B–occurrence of anaerobic sulfur oxidation.

As shown in Fig. 1, changes in sewage temperature influenced the concentrations of reduced sulfur (sulfide) and oxidized sulfur (sulfate) throughout the 2 years of operation of the reactor. After the addition of sodium sulfate, the level of oxidized sulfur increased during low temperature periods, but decreased during high temperature periods. This result suggested that a lower sewage temperature was essential for stimulating the anaerobic sulfur oxidation reaction. This was confirmed in the UASB profiles during the anaerobic sulfur reduction and oxidation periods, as shown in Fig. 2. The UASB profiles showed that, during the high temperature (Fig. 2A), the high sulfate concentration of the UASB influent decreased significantly at the bottom part of the reactor and, thereafter, a low sulfate concentration was observed until the top part of the reactor, whereas the low sulfide concentration increased significantly at the bottom part and was maintained at a high concentration until the top part of the reactor, thereby indicating that the anaerobic sulfur reduction reaction had occurred. During the low temperature (Fig. 2B), a high sulfate concentration in the UASB influent was reduced to sulfide at the bottom part until the middle part of the reactor, whereas the concentration of sulfide at the middle part of the reactor decreased while the sulfate concentration increased, which indicated that the anaerobic sulfur oxidation reaction had occurred.

The average total BOD concentration of the UASB effluent was 85.1 mg L^−1^ with a removal rate of 46.7%, whereas the average total COD concentration of the UASB effluent was 176.4 mg L^−1^ with a 45.5% removal rate. The average pH and ORP during the operational period was 7.0 and −281 mV, respectively. More details on the performance of the UASB reactor are shown in Table 1. The results obtained showed that the total BOD removal rate was significant reduced during the low temperature period, which was consistent with the findings of Singh and Viraraghavan (36). These results also indicated a stable system and were typical for the operation of such a reactor maintained under anaerobic conditions, in which no indications of DO, nitrate, or nitrite were identified. Therefore, contrary to earlier findings (29), the sulfur oxidation reaction in this study occurred in the absence of any electron acceptors.

### Microbial community structure and diversity

In the present study, 259,912 sequence reads were generated from 16 samples collected from the UASB reactor during its 2 years of operation (Table S1). Approximately 11,000–23,000 sequence reads per sample were analyzed. According to the Chao1 estimator, the estimated species increased on average by approximately 5- to 7-fold in the samples. Therefore, the coverage values were relatively low (58–71% coverage), which indicates an underestimation of species richness due to the high microbial diversity that exists in the sludge samples. The PD, Shannon and Simpson diversity indexes, which were applied to compare microbial diversity among the samples, also exhibited a high richness, and evenness in microbial diversity existed across all sludge samples. These results were consistent with previous findings in which microbial diversity was high in sewage sludge samples (37).

**Fig. 3:**
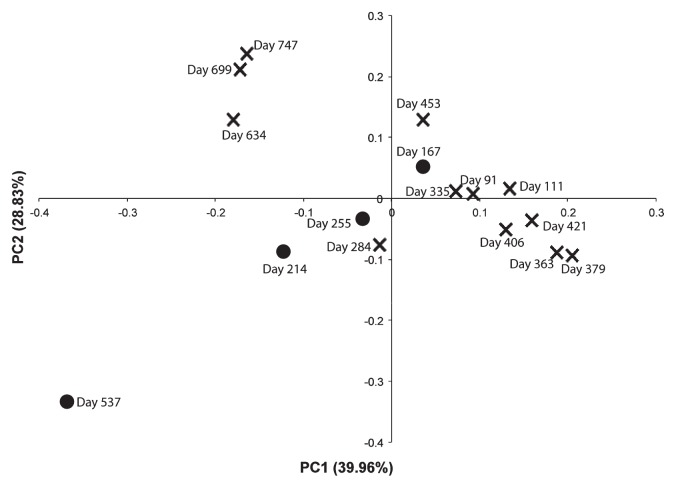
PCoA analysis of UASB sludge samples with weighted UniFrac. ●–oxidation period sludge samples; ×–reduction period sludge samples.

PCoA plot clustering of the phylogenetic diversity (weighted UniFrac distances) of the sludge samples is shown in Fig. 3. PCoA plots showed a distinct overall bacterial community composition in each of the anaerobic sulfur reduction and oxidation periods. The bacterial communities of day 167 from the oxidation period showed similarities between the other bacterial communities from the reduction periods, in which they were clustered together and appeared to exhibit slight changes in microbial activity, which may have been because it was a start-up period. The bacterial communities of day 214 and 255 from the oxidation period were located slightly distant and clustered together, which indicated that their microbial communities had changed during anaerobic sulfur oxidation. The bacterial community of day 284, which was clustered together with those on day 214 and 255, showed that microbial communities had changed after oxidation had ended. After oxidation had completely finished, the bacterial communities of day 335–453 shifted back to the previous cluster, indicating that their microbial communities had reverted back to their initial structure and diversity. The bacterial community of day 537 formed a cluster on its own, which indicated that the bacterial communities on that day differed from the other clusters and that a sudden change had occurred in the microbial communities during anaerobic sulfur oxidation. The microbial communities of day 634–747 showed an obvious shift and formed a distinct cluster, indicating that they were different from the other clusters and that their microbial communities had changed completely after oxidation had ended. The marked differences observed between the anaerobic sulfur oxidation and reduction periods showed that the phylogenetic diversity of the sludge samples was related to microbial communities, which were, in turn, influenced by the temperature and also possible by the surrounding environment.

### Bacterial distribution in UASB sludge

**Fig. 4:**
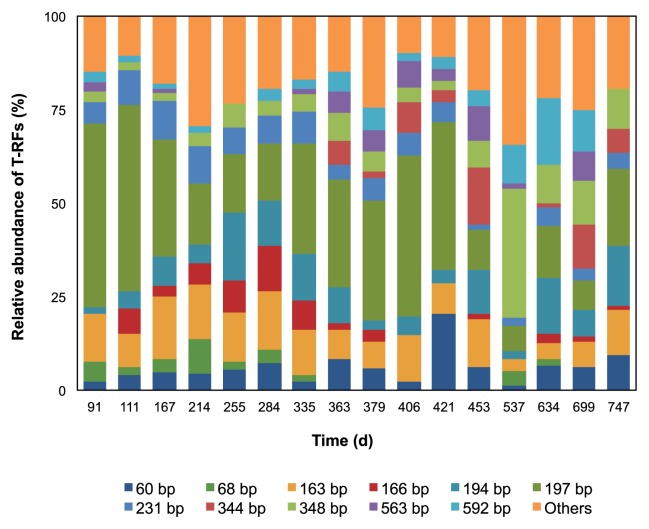
Distribution of different phylogenetic bacteria communities as determined by T-RFLP profiles after *Hha*I digestion of the 16S rRNA gene.

T-RFLP and next-generation sequencing analyses of the bacterial 16S rRNA gene were performed to characterize and identify the microbial communities existing in the UASB reactor. Both analyses were used to increase the reliability of the results and minimize the biases produced by both methods. The distribution of bacterial communities in the sludge samples before and after sodium sulfate was added, as well as during anaerobic sulfur oxidation and non-oxidation, was determined. The T-RFLP results shown in Fig. 4 yielded a large number of T-RFs in all 16 samples and 11 dominant T-RFs were observed. However, during anaerobic sulfur oxidation, a sudden increase was noted in a 348 bp T-RF. Among the dominant T-RFs, only the 60, 163, 194, 197, 231, and 348 bp T-RFs appeared in all samples. The 163, 194, 197, and 231 bp T-RFs were simulated by TRiFLe as genera *Desulfitibacter*, *Singulisphaera*, uncultured bacteria of the phylum *Caldiserica*, and genus *Blastopirellula*, respectively. As for the 60 and 348 bp T-RFs, each of these had two simulated bacteria species, which were genera *Prosthecochloris* and *Treponema* for the 60 bp T-RF and *Desulfomicrobium* and *Desulfovibrio* for the 348 bp T-RF. Some of the T-RFs represented more than one species, as different species were capable of producing the same T-RF length when using a particular enzyme due to the probability of non-unique restriction enzyme cutting sites and their variation across species (22, 34). In addition, the T-RFs predicted *in silico* and those measured *in vivo* mostly differed by a few base pairs (42); thus, the identity of the bacteria in the community could not be analyzed directly. Since it is not possible to accurately quantify the contribution of each of the species to the T-RF, a sequencing analysis was conducted as a means of identity confirmation and precise quantification of the representation of bacteria in the community.

**Fig. 5:**
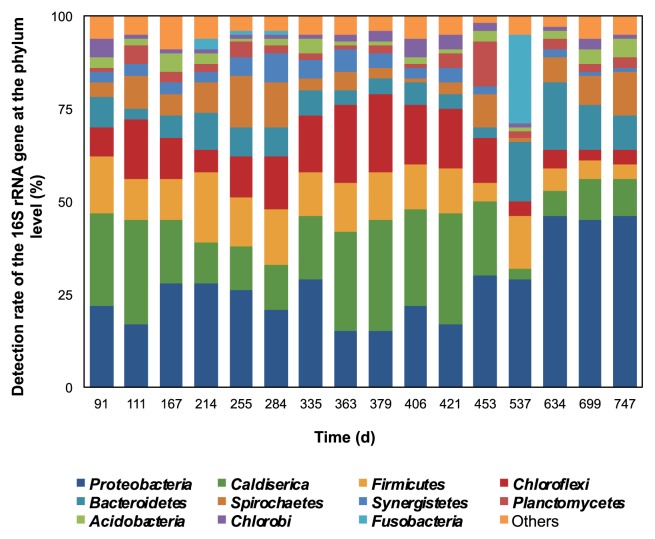
Bacterial community structures of UASB sludge samples at the phylum level.

The sequencing results shown in Fig. 5 showed virtually the same patterns as the T-RFLP results, particularly from day 537 onwards, in which both of the results showed distinctive changes in the microbial community patterns. During the initial stage of anaerobic sulfur oxidation (day 167, 214, and 255), the microbial community patterns were only slightly different from those on the other non-oxidation days, *e.g.* a slight increase was observed in bacteria belonging to the phyla *Proteobacteria*, *Firmicutes*, and *Bacteroidetes*, and a slight decrease in bacteria belonging to the phyla *Caldiserica* and *Chloroflexi*. However, during the second anaerobic sulfur oxidation period (day 537), the microbial community patterns were markedly different from those during the initial oxidation period. The number of representatives of the phyla *Caldiserica* and *Chloroflexi* was markedly lower, whereas that of the phylum *Proteobacteria* was only slightly higher than those in the earlier period. A previous study (1) showed that the uncultured bacteria of phylum *Caldiserica* were the main bacterial group observed in the UASB reactor, which was consistent with the results observed in this study. However, in the initial and second anaerobic sulfur oxidation periods, the number of representatives of the phylum *Caldiserica* decreased. One possible reason is a difference in the primers used in both studies; therefore, a quantitative method such as real-time PCR was needed to clarify these phenomena. The number of bacteria belonging to the phylum *Fusobacteria* was suddenly and markedly higher in the second than in the first oxidation period. Thus, the phylum *Fusobacteria* may only be detectable during the anaerobic sulfur oxidation periods. The microbial community patterns also began to show some changes after day 537, similar to the PCoA analysis (Fig. 3), which may have been due to the adaptation of the microbial communities to the environment.

Overall, the main bacterial groups monitored in the UASB reactor belonged to the phylum *Proteobacteria*, which dominated the UASB sludge samples (27.3±10.4% of the total taxa), followed by the phyla *Caldiserica* (17.8±8.9%), *Firmicutes* (11.3±4.1%), *Chloroflexi* (11.5±6.1%), *Bacteroidetes (*8.0±4.4%), and *Spirochaetes* (6.5±4.0%). Bacteria belonging to the other phyla constituted less than 3% of the total taxa. These bacterial groups were those generally detected in wastewater treatment plants and reactors, particularly representing the phylum *Proteobacteria*, which has been described as the most abundant bacterial group present in reactors (9, 28, 40, 43). Class *Deltaproteobacteria* was the main group within the phylum *Proteobacteria*, accounting for 78.7±16.7% of the total taxa, followed by classes *Alphaproteobacteria (*13.6±9.3%), *Beta-* and *Gammaproteobacteria* (3.2±6.5% and 2.6±3.0%, respectively), and *Epsilonproteobacteria* (1.0±1.7%), which was similar to the microbial community structure previously detected in various types of UASB sludge (28). The major groups within class *Deltaproteobacteria* were genus *Desulfovibrio*, followed by the genera *Desulforhabdus* and *Smithella*. However, no previous studies have mentioned that these bacterial groups are SOB or involved in sulfur oxidation.

The reverse reaction of the anaerobic oxidation of methane (AOM) with sulfate reduction appears to be required for anaerobic sulfur oxidation to occur. Holler *et al.* (17) demonstrated that the reverse direction of AOM with sulfate was catalyzed by AOM consortia. These consortia, which mediate AOM with sulfate, were composed of anaerobic methanotrophic (ANME) archaea and SRB (24). Although ANME-related archaea were not detected in the UASB reactor, methanogenic archaea and SRB were detected, as reported previously by Aida *et al.* (1). This finding suggested that SRB that were present in the UASB reactor acted as the main players in anaerobic sulfur oxidation.

Bacteria belonging to the phylum *Fusobacteria* are obligate anaerobic non-spore-forming Gram-negative bacilli. These bacteria were initially associated with the human mouth and gastrointestinal tract (2, 31), but were subsequently isolated from anoxic sediment and sludge (4, 33), anaerobic mud (20, 41), and cold deep-marine sediment (44), which produce H_2_ and acetate as major fermentation products under different conditions (*e.g.* variations in the carbon source and temperature). Some of these bacteria are psychotropic bacteria with an optimal growth temperature of 18.5°C, while some are mesophilic bacteria with an optimal growth temperature of 28–37°C. Therefore, the uncultured bacteria of the phylum *Fusobacteria* found in this study, which were detected during anaerobic sulfur oxidation, may not be related to any other known bacteria in this phylum and are likely to play a role in anaerobic sulfur oxidation. Nevertheless, further studies are needed to elucidate the function of these uncultured bacteria.

### Influence of environmental conditions on microbial community structure and diversity

**Fig. 6:**
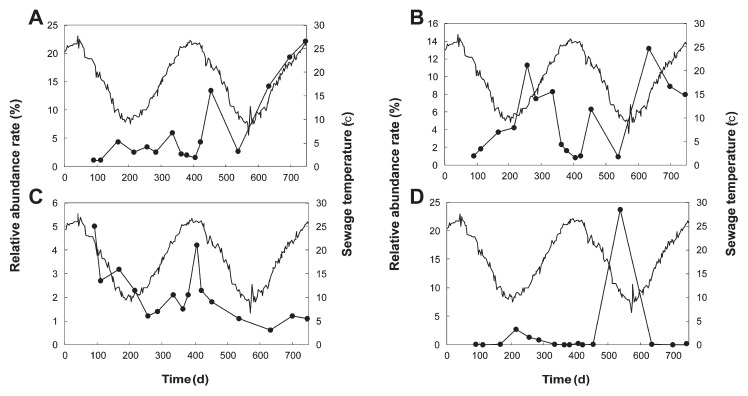
Significant relationships between relatively abundant bacterial groups (●) present in UASB sludge samples and sewage temperature (−) of the UASB reactor. A–*Desulfovibrio*; B–*Desulforhabdus;* C–*Smithella*; D–uncultured bacteria of phylum *Fusobacteria*.

Environmental conditions strongly influence the structure and diversity of microbial communities and are related to the types of reactions that can occur under these conditions. Temperature acclimation, in particular, may alter microbial community activity, structure, and diversity (25, 35). In this study, temperature appeared to influence microbial activity and diversity in the UASB sludge, which then stimulated the sulfur redox reaction to take place. As discussed earlier, anaerobic sulfur oxidation occurred at low temperatures and the main bacterial groups observed during the period of oxidation belong to the genera *Desulfovibrio*, *Desulforhabdus*, *Smithella*, and uncultured bacteria of the phylum *Fusobacteria*. Thus, a correlation was observed between the abundance of the members of these four genera and the temperature in the reactor. The results in Fig. 6 showed that members of the genus *Desulforhabdus* and uncultured bacteria of the phylum *Fusobacteria* were highly abundant when the temperature was low and vice versa. In contrast, the abundance of members of the genera *Desulfovibrio* and *Smithella* was not related to temperature. This result suggested that bacteria belonging to the genus *Desulforhabdus* and uncultured bacte-ria of the phylum *Fusobacteria* were involved in anaerobic sulfur oxidation. Although the genus *Desulforhabdus* is SRB, these bacteria may have a dual function and also play a role in anaerobic sulfur oxidation in the UASB reactor. However, further validation methods such as stable isotope probing and the combination of microautoradiography with fluorescent *in situ* hybridization methods (10, 18) are needed to gain more detailed insights into the physiological properties of these bacteria.

## Conclusion

The microbial diversity analysis performed in the present study yielded a comprehensive overview of the abundance, distribution, characteristics, and phylogenetic diversity of microbes existing in the UASB reactor. A highly diverse bacterial community was present, with the genera *Desulfovibrio*, *Desulforhabdus*, and *Smithella* of the phylum *Proteobacteria* as well as uncultured bacteria of the phylum *Fusobacteria* being the main bacterial groups observed during the period of anaerobic sulfur oxidation. However, only the genus *Desulforhabdus* and uncultured bacteria of the phylum *Fusobacteria* were influenced by temperature, which suggested that these two bacterial groups were involved in the sulfur cycle and played a role in anaerobic sulfur oxidation. The mechanisms or pathways used by these bacteria to oxidize sulfur to sulfate currently remain unknown. Therefore, further analyses are required to provide a clearer and better understanding of anaerobic sulfur oxidation in the UASB reactor.

## Supplementary Information



## Figures and Tables

**Table 1:** Operational conditions and performance of the UASB reactor

Sample (day)	Influent temperature (°C)	Influent pH	Influent ORP (mV)	Sulfide concentration (mg-S L^−1^)		Sulfate concentration (mg-S L^−1^)		Influent nitrate concentration (mg-N L^−1^)	Influent ammonium concentration (mg-N L^−1^)	BODtotal removal rate (%)	CODtotal removal rate (%)
	
				Influent	Effluent	Influent	Effluent				
91	23.5	6.7	−258	5.5	9.6	9.2	1.1	0.0	32.6	93	57
111	18.2	6.9	−224	0.0	19.5	142.4	129.9	0.0	14.8	79	54
167	11.6	6.8	−306	0.0	19.0	92.4	75.4	0.0	23.2	37	27
214	10.9	7.0	−245	0.0	27.2	83.6	44.6	0.0	28.4	31	39
255	12.6	7.3	−258	0.0	32.8	69.1	71.1	0.0	33.2	39	31
284	16.0	7.0	−211	2.2	27.7	41.4	8.6	0.0	43.5	64	32
335	22.9	7.2	−279	4.0	43.0	51.0	1.8	0.0	29.2	51	48
363	26.4	6.8	−278	5.7	39.1	45.0	2.5	0.0	32.4	63	58
379	25.4	6.7	−275	11.3	41.2	47.0	4.2	0.1	26.5	80	78
406	26.2	6.8	−242	4.0	43.1	37.0	2.4	0.2	27.4	84	77
421	26.0	6.8	−369	3.4	23.6	23.0	1.4	0.2	29.1	78	53
453	21.6	7.0	−228	3.1	17.5	38.0	3.3	0.1	41.2	68	52
537	11.7	7.7	−203	1.7	13.5	52.0	43.7	0.3	16.0	18	42
634	14.2	7.4	−206	2.4	31.7	33.8	12.8	0.1	13.2	41	53
699	22.1	6.6	−212	1.3	27.7	12.1	12.8	0.3	32.5	52	40
747	26.0	6.9	−321	9.1	32.0	45.8	4.1	0.1	33.0	66	57
