# Supplementary material for: Diversity Profile of Microbes Associated with Anaerobic Sulfur Oxidation in an Upflow Anaerobic Sludge Blanket Reactor Treating Municipal Sewage
Source: Microbes Environ. 2015 Mar 28;30(2):157–63. doi: 10.1264/jsme2.ME14105 (PMC4462926; doi:10.1264/jsme2.ME14105)
Supplement: Supplementary file 1 [file 30_157_s1.pdf]

Supplemental Material

Table S1. Diversity indices of the UASB sludge samples

Sample (day)	Anaerobic sulfur reduction/oxidation	Number of sequence	Number of OTU	Chao1	Coverage (%)	PD*	Shannon*	Simpson*
91	Reduction	14343	5823	36628	65	258.94	9.16	0.97
111	Reduction	15096	6007	30853	66	245.38	8.97	0.96
167	Oxidation	17752	8156	47198	60	298.38	9.97	0.98
214	Oxidation	21972	10254	63745	60	312.42	10.34	0.99
255	Oxidation	16233	7719	43872	59	294.50	10.21	0.99
284	Reduction	11836	5717	34783	58	289.77	10.11	0.99
335	Reduction	16213	7444	44072	60	291.35	9.93	0.98
363	Reduction	13716	5207	29837	68	235.85	8.91	0.96
379	Reduction	13756	5191	29366	68	233.32	8.69	0.95
406	Reduction	15017	5647	31792	68	241.72	8.97	0.97
421	Reduction	15512	5317	29618	71	219.46	8.48	0.95
453	Reduction	15590	5874	30759	68	231.66	8.74	0.96
537	Oxidation	14001	5988	37038	63	284.03	9.18	0.96
634	Reduction	17354	7740	43674	62	283.90	9.91	0.99
699	Reduction	22886	9205	46527	66	262.72	9.55	0.98
747	Reduction	18635	7690	40045	65	266.06	9.38	0.97

Abbreviation: OTU - Operational taxonomic unit; PD - Phylogenetic diversity
*Calculations determined at 0.03 dissimilarity based on 10,000 reads.
